# Pharmacogenomics-Guided Pharmacotherapy in Patients with Major Depressive Disorder or Bipolar Disorder Affected by Treatment-Resistant Depressive Episodes: A Long-Term Follow-Up Study

**DOI:** 10.3390/jpm12020316

**Published:** 2022-02-19

**Authors:** Antonio Del Casale, Leda Marina Pomes, Luca Bonanni, Federica Fiaschè, Clarissa Zocchi, Alessio Padovano, Ottavia De Luca, Gloria Angeletti, Roberto Brugnoli, Paolo Girardi, Robert Preissner, Marina Borro, Giovanna Gentile, Maurizio Pompili, Maurizio Simmaco

**Affiliations:** 1 Department of Dynamic and Clinical Psychology, and Health Studies, Faculty of Medicine and Psychology, Sapienza University, 00189 Rome, Italy; antonio.delcasale@uniroma1.it (A.D.C.); paolo.girardi@uniroma1.it (P.G.); 2 Unit of Psychiatry, ‘Sant’Andrea’ University Hospital, 00189 Rome, Italy; luca.bonanni4@gmail.com (L.B.); federica.fiasche@uniroma1.it (F.F.); clarissa93.zocchi@gmail.com (C.Z.); alessio.padovano@uniroma1.it (A.P.); gloria.angeletti@uniroma1.it (G.A.); roberto.brugnoli@uniroma1.it (R.B.); maurizio.pompili@uniroma1.it (M.P.); 3 Department of Neuroscience, Mental Health, and Sensory Organs (NESMOS), Faculty of Medicine and Psychology, Sapienza University, 00189 Rome, Italy; ledama@hotmail.it (L.M.P.); ottavia_deluca@yahoo.it (O.D.L.); marina.borro@uniroma1.it (M.B.); giovanna.gentile@uniroma1.it (G.G.); 4 Unit of Laboratory and Advanced Molecular Diagnostics, ‘Sant’Andrea’ University Hospital, 00189 Rome, Italy; 5 Structural Bioinformatics Group, Institute for Physiology, Charité—University Medicine Berlin, 10115 Berlin, Germany; robert.preissner@charite.de

**Keywords:** pharmacogenomics, treatment-resistant depression, mood disorders, major depressive disorder, bipolar disorder

## Abstract

Treatment-resistant depression (TRD) reduces affected patients’ quality of life and leads to important social health care costs. Pharmacogenomics-guided treatment (PGT) may be effective in the cure of TRD. The main aim of this study was to evaluate the clinical changes after PGT in patients with TRD (two or more recent failed psychopharmacological trials) affected by bipolar disorder (BD) or major depressive disorder (MDD) compared to a control group with treatment as usual (TAU). We based the PGT on assessing different gene polymorphisms involved in the pharmacodynamics and pharmacokinetics of drugs. We analyzed, with a repeated-measure ANOVA, the changes between the baseline and a 6 month follow-up of the efficacy index assessed through the Clinical Global Impression (CGI) scale, and depressive symptoms through the Hamilton Depression Rating Scale (HDRS). The PGT sample included 53 patients (26 BD and 27 MDD), and the TAU group included 52 patients (31 BD and 21 MDD). We found a significant within-subject effect of treatment time on symptoms and efficacy index for the whole sample, with significant improvements in the efficacy index (F = 8.544; partial η² = 0.077, *p* < 0.004) and clinical global impression of severity of illness (F = 6.818; partial η² = 0.062, *p* < 0.01) in the PGT vs. the TAU group. We also found a significantly better follow-up response (χ² = 5.479; *p* = 0.019) and remission (χ² = 10.351; *p* = 0.001) rates in the PGT vs. the TAU group. PGT may be an important option for the long-term treatment of patients with TRD affected by mood disorders, providing information that can better define drug treatment strategies and increase therapeutic improvement.

## 1. Introduction

The incidence of major depression is growing each year. A long illness course occurs in approximately 20–30% of patients with major depressive disorder (MDD), leading to a worsening quality of life and a significant increase in health care utilization and treatment costs [1,2,3]. Despite drug therapy being the main type of intervention, the response to first-line antidepressants is moderate (40–60%) and only a limited number of patients achieve remission (30–45%) [4,5,6]. Furthermore, 10–20% of patients have persistent symptoms for two or more years, even with appropriate antidepressant trials, and some disability can persist after symptom remission [7,8,9]. Treating patients with selective serotonin reuptake inhibitors (SSRIs), the most used first-line pharmacological agents, can ensure response rates of up to about two-thirds of patients, with reported remission rates not exceeding one-quarter of patients [10,11,12,13].

There is no universally accepted definition of treatment-resistant depression (TRD) to date. More than 10 different definitions can be found in the literature, with different clinical pictures specifically defined. The most accepted definition of TRD is a depressive episode with a lack of treatment response after administration of two or more trials with antidepressant drugs belonging to different pharmacological classes and administered at appropriate dosages and periods [14,15,16,17,18].

The Sequenced Treatment Alternatives to Relieve Depression (STAR*D) study showed reduced possibilities for improvement after the second treatment failure [5,19]. There is no general agreement to define a minimum number of treatments indicative of resistance, but it is widely accepted that the dose and duration of treatments must be appropriate, and patients must be completely compliant [14,20]. For bipolar disorder (BD), the most common TRD definition requires one prior treatment failure without consensus about the adequacy of either dose or duration [14].

In recent years, research in the medical field has increasingly focused on personalized medicine, trying to identify the most suitable therapy in a certain sense tailored for the patient based on the study of the patient’s genomics. Identifying the genes involved in the response to psychotropic drugs may be useful in improving therapeutic efficacy and therefore clinical outcome [21]. Gene–drug interactions may influence the safety of drugs associated with harmful side effects [22]. Pharmacogenomics might help identify the most suitable and tolerated drugs at the most appropriate dosages. In brief, the main objectives of pharmacogenomics are to individualize the selection of drugs to improve efficacy and safety and reduce costs.

Genome-wide association studies and candidate gene studies have quantified how some genetic factors can influence the response to psychopharmacological therapy. In particular, the response variability of the individual is influenced by genetic factors in 42% of candidates, with the environment and environment–genotype interaction being crucially important [23].

Regarding mood disorders, numerous genes have been studied, such as those encoding proteins involved in the monoamine system (e.g., SLC6A4, HTR1A, HTR2A, MAO, and COMT), glutamatergic system, neuroplasticity (BDNF), inflammation (IL-1¦Â) and the hypothalamic–pituitary–adrenal axis (FKBP5 and NR3C1). The most important genes are involved in the function of the serotonergic system; the HTR2A serotonin receptor gene is one of the most investigated [24,25]. Other genes (GNB3, HTR1B, TPH2, and NET) are involved in the pharmacogenomics of mood disorders [21,26,27].

Genes encoding for cytochrome P450 enzymes (CYP2B6, CYP2D6, CYP2C9, and CYP2C19) and ATP-binding cassette subfamily B member 1 (ABCB1) are involved in the pharmacokinetics of drugs and drug resistance. CYP2C19 and CYP2D6 encode two Phase I metabolism enzymes that are critically important in the metabolism of most SSRIs and all tricyclic antidepressants. Variations of these genes differentiate an individual’s metabolizer status (i.e., poor, intermediate, normal, rapid, and ultra-rapid) according to published protocols [28,29,30]. These alternative forms can affect the response to antidepressant therapy [31,32,33,34]. Drug efflux pump ABCB1 plays a crucial role in the absorption and distribution of several antidepressant medications; the genetic variants are designated as rs1045642 (C3435T) and rs2032582 (G2677T), for which the TT genotype is associated with lower substrate efflux and higher tissue concentrations compared to alternative genotypes [35], although more information is needed [36,37]. The Genomics Used to Improve DEpression Decisions (GUIDED) study showed that the clinical response and remission rates for patients with MDD treated with combined multi-gene pharmacogenomics-guided therapy (PGT) were significantly higher compared to those with the standard treatment, especially when the drugs used were congruent with the patients’ genetic profile [38].

On this basis, we hypothesized that PGT, compared to treatment as usual (TAU), would be followed by a better symptom improvement in TRD, both in MDD and BD. The main objectives of our study were to assess whether patients with MDD and BD who have undergone PGT for TRD achieve clinical improvement and show better tolerability of drugs.

## 2. Materials and Methods

An observational, retrospective study was conducted during the years 2018–2021 at the Centre of Personalized Medicine and Service of Personalized Mental Health and Pharmacogenomics, Unit of Psychiatry, Sant’Andrea University Hospital, Sapienza University, Rome. We obtained written consent from all participants after fully informing them about the type and aims of the treatment. This study was approved by the local ethical committee (prot. N. 6279/2021) and was carried out following the Principles of Human Rights adopted by the World Medical Association (WMA) at the 18th WMA General Assembly, Helsinki, Finland, in June 1964, and subsequently amended by the 64th WMA General Assembly, Fortaleza, Brazil, in October 2013.

Exclusion criteria included minors (≤18 years) or those of advanced age (≥75 years), incongruent psychotropic drugs at baseline or during the study period, substantial changes in pharmacotherapy during the study, concurrent substance use disorders (except nicotine dependence), neurological conditions (epilepsy, major neurocognitive disorders, Parkinson’s disease, and Huntington’s chorea), and severe acute organic illnesses (major cardiovascular pathologies, uncontrolled diabetes, serious toxic, infectious and metabolic diseases, malignancy, liver failure, and renal failure).

A group of patients underwent a pharmacogenomics assessment for severe treatment-resistant depression with manifestation of side effects, and another group of patients followed a standard treatment. We subdivided those patients into two groups (PGT and TAU). At the baseline assessment, all the patients had been on medications for at least 12 months, with two or more subsequent different standard antidepressant treatments to which they were poorly responsive or unresponsive (less than a 50% drop on the Hamilton Depression Rating Scale (HDRS)). We subsequently assessed the clinical characteristics of the sample after six months from baseline.

### 2.1. Assessment of Pharmacogenes with Drug-PIN Software

All patients were genotyped for 108 polymorphisms in 48 pharmacogenes, including Phase I and Phase II drug-metabolizing enzymes, drug transporters, and targets (see Supplementary Table S1), using a custom panel designed for targeted DNA re-sequencing via Ion AmpliSeq™ Library Kit 2.0 chemistry and next-generation sequencing platform Ion Chef/Ion S5 system (Thermo Fisher Scientific, Waltham, MA, USA) according to the manufacturer’s instructions. DNA samples were obtained from 5 mL of peripheral blood using a QIAsymphony automatic system for nucleic acid extraction (QIAGEN, Hilden, Germany).

Drug-PIN software (https://www.drug-pin.com/, last accessed on 15 December 2021) is an integrated bioinformatics solution that allows automatic interpretation of patient data to be correlated to an individual response to drugs, including age, habits (alcohol, caffeine, and cigarette consumption), hepatic/renal function and genomic profile. Included data are combined to give a drug–drug and drug–gene interaction profile for one or more proposed medications. Drug-PIN software associates a numerical score to a proposed therapeutic scheme that reflects the theoretical therapeutic index (tolerability/efficacy): the greater the associated number, the worse the proposed scheme.

According to the case under examination, the Drug-PIN score has different components: a percentage inherent to the drug–drug interactions known in the literature; a portion related to the patient’s age, which considers the PRISCUS list that assesses the adequacy of a large panel of medicaments in the elderly; a percentage inherent to the renal function, which is calculated by following the indications of appropriateness dictated by the Beers list; and a portion related to pharmacokinetic problems and calculated based on the genotype of the inserted polymorphisms (drug–gene interactions).

The terms under which each of these rates forms the final score are clarified in a report and alerts related to the detection of inappropriate or unfavorable drug interactions. Drug-PIN software allows the user to choose between the best theoretical therapeutic options by supporting the construction of new drug combinations: it automatically assesses (each time recalculating the score) the impact of a new drug inserted in polypharmacy, providing a view of the best hypothetical alternatives (greater safety and efficacy) for each class of drugs (e.g., antidepressants, benzodiazepines, antipsychotics, and mood stabilizers). Recommendations were based on a drop in the Drug-PIN score related to the prescribed benzodiazepines, antidepressants, antiepileptics, and antipsychotics.

### 2.2. Medication Congruence with Pharmacogenomics Testing

Consistent with previous studies, we considered the prescribed drugs congruent with the pharmacogenomics test if they belonged to the use-as-directed or use-with-caution categories according to the Drug-PIN score reduction [39,40]. Conversely, we considered prescribed medications to be incongruent if they were not recommended according to the Drug-PIN based assessment for drug–drug and/or drug–gene interactions. In this case, the patients were excluded from the study, being considered as dropout subjects.

### 2.3. Psychiatric Assessments

We analyzed the patients’ medical records included at baseline and the 6 month follow-up. We diagnosed patients based on DSM-5 criteria [41].

The clinical status of the patients was assessed with the Clinical Global Impression (CGI) scale [42]. The CGI severity of illness is rated on a seven-point scale, with a range of responses from 1 (normal) through 7 (the most severely ill patients). The CGI change scores range from 1 (very much improved) through 7 (very much worse). The CGI efficacy index (CGI-EI) ratings take into account both therapeutic efficacy and treatment-related adverse events and range from 0 (marked improvement and no side effects) to 16 (unchanged or worse, with side effects outweighing therapeutic effects). We used the CGI-EI as a measure of the patients’ tolerability of the drugs, assessing the therapeutic effect of treatment with psychiatric medication and associated side effects.

The 17-item HDRS [43] was used to assess depressive symptoms. The HDRS is a clinician-rated scale designed to assess the severity of depressive symptoms: single items are rated on a Likert scale of 0–4 (8 items) or 0–2 (9 items). The total score is calculated on the first 17 items: 0–7, normal; 8–16, mild depression; 17–23, moderate depression; 24 or more, severe depression [44]. A score of 7 or less indicates remission of symptoms. We assessed adverse events at follow-up, in which we investigated the presence of adverse events and eventual correlations with the drug treatment. We summarized the overall study design in Table 1.

### 2.4. Statistical Analysis

We compared the baseline sociodemographic and clinical characteristics of the two groups of patients (PGT and TAU) using one-way ANOVA for continuous variables (e.g., age, CGI, and HDRS values) and the Pearson chi-squared test for categorical variables (e.g., sex, response, and remission). We measured HDRS score changes between the baseline and the 6 month follow-up. We considered the standard for follow-up response a 50% drop in HDRS, and the standard for remission an HDRS score of 7 or less. We performed the Kolmogorov–Smirnov test, which showed a normal value distribution at each time point. Changes were then analyzed by repeated-measures ANOVA, with the treatment group as the between-subject factor and treatment time (baseline—6 months) as the repeated-measures factor. Sphericity assumption was tested with Mauchly’s test, which did not show any significance. The cutoff for statistical significance was set at *p* < 0.05 and all *p*-values were two-tailed. We used IBM SPSS Statistics 25.0 (IBM Corporation, 2019, Armonk, New York, NY, USA) software for all analyses.

## 3. Results

### 3.1. Sample Description

We recruited a sample of 111 admitted outpatients with TRD. A total of six patients dropped out of the study because of refusal of the new proposed treatment (three), choice of another type of therapy (one), and non-attendance at follow-up visits (two).

Hence, our final sample included 105 patients (50 men and 55 women) with severe TRD between January 2018 and October 2021. The PGT sample included 53 patients (26 BD and 27 MDD; mean age = 45.21, SD = 16.26), and the TAU group included 52 patients (31 BD and 21 MDD; mean age = 50.79, SD = 14.12).

All patients tolerated the drug treatments without severe complications or hospitalization. No patient manifested clinical hypomanic or manic episodes during the 6 month study period. The study’s baseline sociodemographic characteristics and mean total HDRS and CGI scores are summarized in Table 1. The PGT vs. TAU group did not differ significantly in age (F = 3.520; *p* = 0.063) or sex composition (χ² = 1.6020; *p* = 0.206) and mean baseline values of the HDRS (F = 0.437; *p* = 0.510), but significantly differed in CGI-EI scores (F = 24.917; *p* < 0.001) (Table 2).

We summarize the changes in drug treatment at the study baseline in Table 3.

### 3.2. Treatment Effects

**Figure 1:**
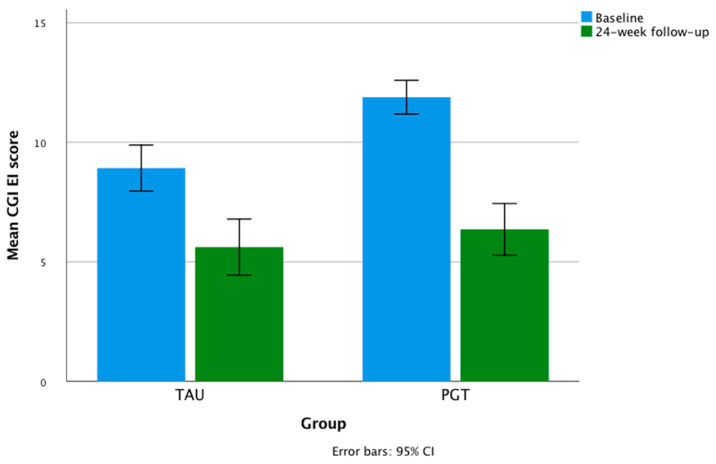
Mean changes in CGI-EI scores during the study.

**Figure 2:**
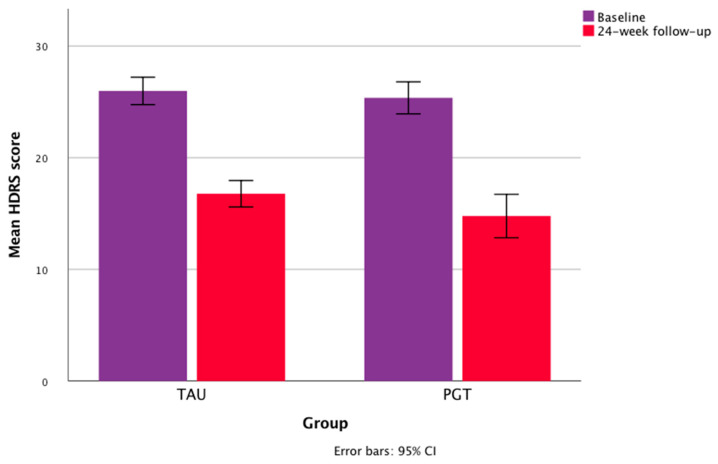
Mean changes in HDRS scores during the study.

At a 24 week follow-up, the mean drop in the HDRS values in TAU was 35.2%, and 42.87% in PGT. Repeated-measures ANOVA showed a significant within-subject effect of treatment time in the whole sample for the HDRS scores (F = 400.789; partial η² = 0.796; *p* < 0.001) and CGI-EI (F = 135.280; partial η² = 0.568; *p* < 0.001), with a significant within-subject time × group (PGT vs. TAU) interaction for the CGI-EI (F = 8.544; partial η² = 0.077, *p* = 0.004) and CGI-S (F = 6.818; partial η² = 0.062, *p* < 0.01) scores, and no significant time × group interaction for the HDRS values (F = 1.929; partial η² = 0.018, *p* = 0.168). The univariate tests showed no significant within-subject time × organic comorbidity and time × diagnosis interactions on the HDRS and CGI-EI scores (Table 4). The mean changes in total HDRS and CGI scores over time are shown in Figure 1 and Figure 2.

### 3.3. Response/Remission Rates at Follow-Up Points

In the TAU group, the follow-up response was 17.3% and the remission rate was 3.8%, whereas the PGT group showed a follow-up response of 37.7% and a remission rate of 26.4%. We found a significant between-group difference in the follow-up response rate (χ² = 5.479, *p* = 0.019) and remission rate (χ² = 10.351, *p* = 0.001). The response and remission rates according to sex and diagnosis are shown in Table 5.

## 4. Discussion

Our study findings showed that PGT of TRD in patients with MDD or BD was followed by good long-term response and remission rates. They also highlighted significantly better tolerability of drugs in patients who underwent PGT.

We found a response rate of 17.3% after 6 months of TAU. The TRD response rate that we found is comparable to the rates for standard drug treatment, although there are no such long-term follow-up studies. Previous studies on standard-treated patients with TRD have reported response rates of approximately 16–28% and remission rates of approximately 10–13% after 4–8 weeks [5,38]. The lack of remission in our TAU sample may be due to the long-term follow-up assessment.

Our results are consistent with those of the GUIDED study, which demonstrated the clinical utility of pharmacogenomics in 1167 patients with MDD suffering a moderate to very severe depressive episode [38].

We found a response rate of 37.7% and a remission rate of 26.4% after 6 months of PGT. Furthermore, our PGT sample showed a 42.87% drop in the HDRS mean score from baseline to the 6 month follow-up, which is consistent with the 40.5% decrease in the HDRS score after 2 months of PGT in patients with TRD reported in the study by Altar et al. [39].

In our sample, the response and remission rates appear to be promising, despite the difficulties in treating this challenging patient population, who exhibited depressive episodes resistant to various previous pharmacological treatments, different schemes, and therapeutic approaches. Our results reinforce the evidence that the treatment approach guided by pharmacogenomics might have greater efficacy in the long term in patients with TRD [44].

We found that PGT was followed by both symptom improvement and better tolerability of drugs compared to standard treatments. This is in line with previous evidence for the impact of genomics on antidepressant response. Although there is inconsistent evidence [45,46], PGT may help to predict and avoid well-known possible gene–drug adverse interactions. For example, the S allele of 5-HTTLPR has been generally related to greater adverse drug reaction burden during SSRI treatment, particularly regarding antidepressant-induced mania, gastrointestinal adverse events [47], and headache [48]. The same S allele was related to greater early side effects and poorer early response of psychosis symptoms to citalopram and risperidone in dementia [49]. Among paroxetine-treated elderly patients with MDD, S allele carriers showed more severe adverse events, tolerated lower daily doses, and had more treatment discontinuations [50]. Other important associations include MC4R genetic variants with atypical antipsychotic-induced weight gain [51] and variants of genes encoding for cytochrome P450 enzymes (mainly CYP2D6 and CYP2C19) with a side effect burden of drugs [52]. The results of better tolerability of our PGT sample could be ascribed to having considered the genomics of the serotonin transporter and other proteins involved in the pharmacokinetics and pharmacodynamics of psychotropic medications.

Drug-PIN software analysis, based on the functional biochemical profile of each patient, assesses multiple gene–drug interactions that can affect the safety and efficacy of psychoactive drugs. The same analysis also considers drug–drug interactions in patients treated with combinations of drugs. Multiple interaction analysis is crucial, considering that scientific evidence concerning single-gene tests is often ineffective in achieving a therapeutic response [53]. Some randomized studies have shown that selecting antidepressants guided by multi-gene pharmacogenomics tests is useful in obtaining a pharmacological response [54,55,56]. In addition, a significant improvement in depressive symptoms with PGT has been reported by two open-label, non-randomized studies of MDD without TRD [57,58].

Compared to existing studies, the innovative evidence of our study is the long-term (24 week) maintenance of the overall response to antidepressant treatment and the good response to PGT in both MDD and BD patients with TRD.

Attention should be focused on the response and remission outcomes that we found for TRD in patients with BD who followed PGT, which showed similar rates for TRD in patients with MDD and BD. This finding is in line with evidence of reduced admission to emergency rooms for patients with BD after the introduction of PGT, with the consequent significant savings in healthcare costs [59]. Our data also demonstrated similar responses in patients with and without organic comorbidities.

Our results need to be replicated as no other long-term follow-up studies have focused on PGT of bipolar TRD. New information and PGT implementation may derive from the study of sociodemographic, ethnic, biological, and psychological characteristics of patients with BD and may be related to the manifestation of mood disorders, suicidality, and clinical responses to antipsychotics, antiepileptics, and lithium [60,61,62].

## 5. Limitations

The main limitation of this study is the small sample size and its retrospective nature, for which the results should be interpreted with caution and need to be replicated in larger, prospective, and multicenter studies.

## 6. Conclusions

Our study findings showed that PGT of TRD in patients with MDD or BD was followed by good long-term response and remission rates compared to standard treatments. We highlighted better tolerability of drugs and better improvement of symptoms based on the physician impression of patients who underwent a guided treatment. The response and remission outcomes and tolerability variables showed similar rates for patients with MDD and BD. PGT may be an essential option for the long-term treatment of patients with TRD affected by mood disorders and can give information for better drug treatment strategies and therapeutic improvement.

In recent years, the evidence for applying PGT in MDD and BD has been growing, although more studies on drug safety, side effect burden, and treatment adherence are needed.

The use of bioinformatics tools, allowing rapid and reliable evaluation of many pharmacogenomics variables and drug–drug interactions, can help to systematically apply the possibilities of choosing personalized drug treatments based on pharmacogenomics screening with better theoretical efficacy and tolerability in a real-world clinical setting.

## Figures and Tables

**Table 1:** Overall study design.

Patients with TRD	
PGT	TAU
Baseline: Psychiatric and psychometric assessment Assessment of pharmacogenomics interaction Clinical guidelines-based treatment	Baseline: Psychiatric and psychometric assessment Clinical guidelines-based treatment
6 month follow-up: Psychiatric and psychometric assessment	6 month follow-up: Psychiatric and psychometric assessment

**Table 2:** Sociodemographic and clinical characteristics of the sample.

Variable	PGT Group (*n* = 53)	TAU Group (*n* = 52)	1-Way ANOVA Pearson χ²	*p*
Mean age, years (SD)	45.21 (16.26)	50.79 (14.12)	F = 3.52	0.063
Sex, women/men ratio	31/22	24/28	χ² = 1.602	0.206
Diagnosis (BD/MDD)	26/27	31/21	χ² = 1.179	0.278
Mean baseline HDRS score (SD)	25.36 (5.2)	25.98 (5.34)	F = 0.437	0.51
Mean baseline CGI-S score (SD)	4.96 (0.78)	5.08 (0.74)	F = 0.596	0.442
Mean baseline CGI-I score (SD)	3.64 (0.79)	3.46 (0.67)	F = 1.589	0.21
Mean baseline CGI-EI score (SD)	11.89 (2.57)	8.92 (3.46)	F = 24.917	***<0.001***

BL: baseline; BD: bipolar disorder; CGI-EI: Clinical Global Impression—Efficacy Index; CGI-I: Clinical Global Impression—Improvement scale; CGI-S: Clinical Global Impression—Severity scale; MDD: major depressive disorder; PGT: pharmacogenomics-guided treatment; SD: standard deviation; HDRS: Hamilton Depression Rating Scale; Bold italics: significant for *p* < 0.05.

**Table 3:** Changes in drug treatment for each study sample.

Previous Drug Treatment	TAU	PGT
Benzodiazepines	75%	50.9%
Antidepressants	86.5%	67.9%
Atypical antipsychotics	73.1%	56.6%
Typical antipsychotics	21.2%	17%
Antiepileptics	69.2%	39.6%
Lithium	63.5%	41.5%
**Baseline Drug Treatment**	**TAU**	**PGT**
Benzodiazepines	53.8%	45.3%
Antidepressants	75%	60.4%
Atypical antipsychotics	53.8%	60.4%
Typical antipsychotics	9.6%	6.4%
Antiepileptics	44.2%	35.8%
Lithium	59.6%	47.2%

**Table 4:** Repeated-measures ANOVA.

Descriptive Statistics		Group	Mean		SD	*n*
HDRS mean score, baseline		TAU	25.9808		4.40361	52
		PGT	25.3585		5.20390	53
		Total	25.6667		4.81118	105
HDRS mean score, 6 month FU		TAU	16.7692		4.25932	52
		PGT	14.7736		7.05375	53
		Total	15.7619		5.89740	105
CGI-S, baseline		TAU	5.0769		0.73688	52
		PGT	4.9623		0.78354	53
		Total	5.0190		0.75931	105
CGI-S, 6 month FU		TAU	3.5577		0.95821	52
		PGT	2.8868		1.47623	53
		Total	3.2190		1.28588	105
CGI-I, baseline		TAU	3.4615		0.67043	52
		PGT	3.6415		0.78677	53
		Total	3.5524		0.73355	105
CGI-I, 6 month FU		TAU	2.2115		0.97692	52
		PGT	2.3585		1.09359	53
		Total	2.2857		1.03510	105
CGI efficacy index, baseline		TAU	8.9231		3.45756	52
		PGT	11.8868		2.56950	53
		Total	10.4190		3.37349	105
CGI efficacy index, 6 month FU		TAU	5.6154		4.21554	52
		PGT	6.3585		3.92279	53
		Total	5.9905		4.06792	105
**Tests of Within-Subjects Contrasts**						
**Source**		**Mean Square**	**F**	***p***	**Partial Eta Squared**	**Observed Power**
Time	HDRS	5143.210	400.789	***<0.001***	0.796	1.000
	CGI-S	169.584	284.763	***<0.001***	0.734	1.000
	CGI-I	84.205	121.724	***<0.001***	0.542	1.000
	CGI-EI	1024.639	135.280	***<0.001***	0.568	1.000
Time × Group (PGT vs. TAU)	HDRS	24.753	1.929	0.168	0.018	0.280
	CGI-S	4.061	6.818	***0.01***	0.062	0.735
	CGI-I	0.014	0.021	0.886	0.000	0.052
	CGI-EI	64.715	8.544	***0.004***	0.077	0.825
Time × Organic comorbidities	HDRS	9.960	0.768	0.383	0.007	0.768
	CGI-S	0.988	1.580	0.212	0.015	0.238
	CGI-I	2.931	4.418	***0.038***	0.041	0.549
	CGI-EI	30.719	3.886	0.051	0.036	3.886
Time × Diagnosis	HDRS	22.745	1.770	0.186	0.017	1.770
	CGI-S	0.003	0.005	0.945	0.000	0.051
	CGI-I	0.062	0.09	0.765	0.001	0.06
	CGI-EI	14.436	1.791	0.184	0.017	1.791

CGI-EI: Clinical Global Impression—Efficacy Index; CGI-I: Clinical Global Impression—Improvement scale; CGI-S: Clinical Global Impression—Severity scale; FU: follow-up; HDRS: Hamilton Depression Rating Scale; PGT: pharmacogenomic-guided treatment; SD: standard deviation; TAU: treatment as usual; Bold italics indicates significant results (*p* < 0.05).

**Table 5:** Response and remission rates in the study sample at 24 week follow-up.

	Response at Follow-Up	Remission at Follow-Up
All patients (rate)	27.6%	15.2%
TAU	17.3%	3.8%
PGT	37.7%	26.4%
Pearson chi-squared	5.479	10.351
*p*	***0.019***	***0.001***

PGT: pharmacogenomic-guided treatment; TAU: treatment as usual; Bold italics: significant for *p* < 0.05.

## Data Availability

Data supporting reported results can be requested from the corresponding author.

## Supplementary Materials

The following supporting information can be downloaded at: https://www.mdpi.com/article/10.3390/jpm12020316/s1. Table S1: Considered pharmacogenes and polymorphisms.
