# Supplementary material for: Pharmacogenomics-Guided Pharmacotherapy in Patients with Major Depressive Disorder or Bipolar Disorder Affected by Treatment-Resistant Depressive Episodes: A Long-Term Follow-Up Study
Source: J Pers Med. 2022 Feb 19;12(2):316. doi: 10.3390/jpm12020316 (PMC8874425; doi:10.3390/jpm12020316)
Supplement: Supplementary file 1 [file jpm-12-00316-s001.zip › Suppl Table 18.02.2022.pdf]

Supplementary Material

Tabel S1 Considered pharmacogenes and polymorphisms

Gene	Polymorphism
5-HTT	5HTT-LPR
ABCB1	rs1128503, rs1045642, rs4148738
ABCC1	rs45511401
ABCC2	rs8187710, rs17222723, rs717620
ABCG2	rs2231142
APOA1	rs1799837
APOB1	rs5742904
CAT	rs1001179
CES1	rs2244613, rs8192935
CETP	rs1532624
COMT	rs4680, rs4633, rs4818
CYBA	rs9932581, rs4673, rs1049255
CYP1A1	rs1048943
CYP1A2	rs2069514, rs762551
CYP2A6	rs28399433, rs1801272
CYP2B6	rs2279343, rs3745274, rs3211371, rs28399499
CYP2C19	rs6413438, rs12248560, rs4244285, rs4986893, rs28399504, rs56337013, rs72558186
CYP2C8	rs11572103, rs1058930, rs72558195
CYP2C9	rs1799853, rs1057910
CYP2D6	rs1065852, rs28371706, rs16947, rs61736512, rs1080985, rs35742686, rs3892097, rs28371725, rs5030655, rs5030867, rs5030656, rs72549351, rs72549354, Gene deletion, Gene duplication
CYP3A4	rs2740574, rs35599367
CYP3A5	rs776746
DRD2	rs1800497, rs1799732, rs1801028
DRD3	rs6280
ENOS3	rs1799983, rs2070744
EPHX1	rs2234922, rs1051740
GPX1	rs1800668
GSTM1	Gene deletion
GSTP1	rs1695, rs1138272
GSTT1	Gene deletion
HMGCR	rs3761740, rs5908
HTR2A	rs6314, rs7997012, rs6311
HTR2C	rs6318
MPO	rs2333227
NAT1	rs5030839, rs56172717, rs56379106, rs4986782
NAT2	rs1801280, rs1799930, rs1799931
NQO1	rs1800566, rs2917666

OGG1	rs1052133
OPRM1	rs1799971
PON1	rs705379, rs662
SLC15A2	rs2257212
SLCO1B1	rs4363657, rs4149056
SOD2	rs4880
TPMT	rs1800462, rs1800460, rs1142345
UGT1A1	rs8175347
UGT2B17	Gene deletion
VDR	rs1544410, rs731236, rs7975232, rs4516035
VKORC1	rs9923231, rs9934438
